# Third-Party Punishment or Compensation? It Depends on the Reputational Benefits

**DOI:** 10.3389/fpsyg.2021.676064

**Published:** 2021-05-28

**Authors:** Zhuang Li, Gengdan Hu, Lei Xu, Qiangqiang Li

**Affiliations:** ^1^Department of Psychology, School of Humanities, Tongji University, Shanghai, China; ^2^School of Medicine, Shanghai Pudong New Area Mental Health Center, Shanghai, China; ^3^Electrical College, Shanghai Dianji University, Shanghai, China

**Keywords:** third-party fairness maintenance, fourth party, reputation, punishment, compensation

## Abstract

Third-party fairness maintenance could win some reputational benefits, and it includes two methods: punishment and compensation. We predicted that the third parties' preference between punishment and compensation are affected by whether they are free to choose between the two methods, and the affection could be interpreted through reputational benefits. The present study includes two sections. In Study 1, the participants acted as fourth parties who were asked to rate the reputations of the third parties who had chosen different response methods to an unfair result of the dictator game. The results showed that (1) there was no reputational difference between the two methods when third parties were not free to choose, (2) but the reputation of compensation was better when third parties were free to choose. In Study 2, the participants acted as third parties. The participants were asked to choose a method to respond to an unfair result of the dictator game. There were two reputational contexts: secret and open. The results showed that (1) when third parties were not free to choose, they had no preference between the two methods under the two reputational contexts, (2) but when third parties were free to choose freely, they prefer punishment under the secret context but prefer compensation under the open context. This study systematically reveals a reputational interaction between fourth and third parties, and verifies the affection of reputational benefits on the third parties' preference between punishment and compensation.

## Introduction

Fairness is an important social rule. Many studies have found that to maintain fairness, people often intervene in unfair events to which they are not connected, and this is referred to as third-party fairness maintenance (Fehr and Gächter, 2002; Fehr and Fischbacher, 2003, 2004; Kroupa, 2014; Hu et al., 2015; Liu Y. et al., 2019). Compared with second-party fairness maintenance (Yamagishi et al., 2009), third-party fairness maintenance would be more neutral and impartial (Bendor and Swistak, 2001). Thus, in our everyday lives, it is often the third parties (such as the police and the courts) who typically take the responsibility of maintaining fairness.

### The Reputational Benefit of Third-Party Fairness Maintenance

Third-party fairness maintenance is beneficial for a group's interests (Fehr and Gächter, 2002). However, there is a dilemma that cannot be ignored: it will lead to an individual cost. If such dilemma continues, third parties involved in fairness maintenance would eventually be eliminated in the process of evolution, but in fact, third-party fairness maintenance is widespread in both the laboratory and the field (Fehr and Gächter, 2002; Fehr and Fischbacher, 2003, 2004; Kroupa, 2014; Hu et al., 2015; Liu Y. et al., 2019). What, therefore, is the motivation for and evolutionary mechanism of third-party fairness maintenance? The indirect reciprocity theory holds that people pay some price to maintain fairness because doing so would win them a good reputation, which will bring some benefit to them in future interactions, and the reputational benefits may cover the costs. This thereby improves the adaptability of third parties (Alexander, 1986; Fehr and Fischbacher, 2003; Bereczkei et al., 2007). Empirical studies have also found that, when in the face of unfair events, compared to bystanders, third parties involved in fairness maintenance are evaluated as more trustworthy, have more opportunities to be chosen as partners, receive more material rewards than bystanders, and are more likely to be chosen as leaders (Barclay, 2006; Santos et al., 2013; Gordon et al., 2014; Raihani and Bshary, 2015a; Jordan et al., 2016).

The premise that third-party fairness maintenance can bring indirect reciprocity is due to the reputation. Whether third-party fairness maintenance results in one having a good reputation largely depends on oberservers. Only when third-party fairness maintenance is seen by fourth-party observers can it win a good reputation. It has been found that when there are audience present, and even only when experimenters were present, third-party fairness maintenance increases (Kurzban et al., 2007). On the contrary, under the condition of secrecy or anonymity, third-party fairness maintenance decreases (Burnham, 2003; Piazza and Bering, 2008). These findings indicate that the reputational context is an important factor that can affect whether a third party would intervene in unfair events.

### Third-Party Punishment and Compensation

In the face of unfair events, third-party fairness maintenance has two intervention methods: punishment and compensation. Punishment is aimed at violators and related to anger (Fehr and Gächter, 2002; Lotz et al., 2011; Liu et al., 2017; Rodrigues et al., 2018), while compensation is aimed at victims and related to compassion (Condon and Desteno, 2011; Leliveld et al., 2012; Sierksma et al., 2014; Hu et al., 2015; Rodrigues et al., 2018). These methods both indicate a third party's concern for fairness and group interests, and both would lead to some good reputation, but there are still some reputational differences between the two methods.

The reputation of punishment may be more complicated than that of compensation. Barbary (2006) divided reputation into four subdimensions: niceness, trustworthiness, group concernedness, and respectability. He found that compared with non-interveners, punishers tended to be better evaluated in the latter three dimensions but were considered less nice. In addition, not all punishments win a good reputation; only those punishments that had been applied against violators would lead to a good reputation. Punishments against cooperators would lead to a bad reputation; however, all forms of compensation, even those for non-victims, would lead to a good reputation (Ozono and Watabe, 2012). Gordon et al. (2014) found that only a dominant punisher can obtain a good reputation because a dominant punisher can prevent revenge.

In addition, many studies have found that, compared with punishment, compensation may be more easily rewarded. For example, Raihani and Bshary (2015a) found that compared with non-intervention, third-party punishment can lead to monetary rewards, but third-party compensation can be rewared more. In economic activities, when interacting with punishers, people are more willing to expect to receive resources from punishers rather than providing resources to them (Horita, 2010). Ozono and Watabe (2012) had made similar observations. They provided their participants with three candidates as partners in the economic game: the punisher, the compensator, and the non-intervener. It was found that people preferred to choose the compensator and non-intervener as partners rather than the punisher. In the dictator game, when the participants were dictators, they allocated less money to the punisher but allocated more to the compensator.

Why are there differences between the two methods regarding reputational benefits? Punishment and compensation not only convey the third parties' concern about fairness but also convey the information about their characteristic traits. Punishment can give people an impression of emotional instability and irritability, which can lead to others becoming feared; however, compensators can make people feel gentle and empathetic (Gordon et al., 2014; Kroupa, 2014; Raihani and Bshary, 2015b). Clearly, the latter would be more preferable. In addition, people need to infer the motivation for third-party fairness maintenance according to the intervention method that had been applied. Only when motivation is really out of concern for fairness and group interests can people provide good reputational feedback for third parties (Raihani and Bshary, 2015b). Punishment may be due to the emotional venting of third parties, which would greatly reduce their altruistic attributes, and then negatively impact their reputations.

How should people infer the characteristic traits and motivation behind the two intervention methods of punishment and compensation? Whether third parties are free to choose between these methods may provide a clue. When exploring punishment and compensation, researches usually involve two types of settings: one is that a third party chooses between punishment, compensation, and keep (i.e., to not intervene); the other is that they choose between punishment and keep or between compensation and keep (Chavez and Bicchieri, 2013; Rodrigues et al., 2018; Liu, Zheng, and Guo, 2019). In the former case, third parties are free to choose between the two intervention methods, while in the latter case, they are not. When third parties are not free to choose, both the methods indicate a third party's willingness to maintain fairness and a third party's concern for fairness and group interests; however, it does not reflect the third party's preference between the two intervention methods. However, when third parties can freely choose, their choices can reflect their preference between the two methods and further reflect their characteristic traits and true motivation. Therefore, we put forward the following as our first hypothesis:

H1: When third parties are unable to freely choose between punishment and compensation, there is no difference in the reputation obtained between the two methods; however, when third parties are able to freely choose, the reputation of compensation is better than that of punishment.

Whether or not one csan freely choose between punishment or compensation cannot only affect the reputation of the two methods but also the third parties' choice between the two methods. Chavez and Bicchieri (2013) found that when third parties can only punish, third parties tend to punish, but when third parties can choose between punishment and compensation, third parties tend to compensate. It is thus evident that third parties intentionally conduct reputation management by choosing between these two intervention methods. When punishment is the only way, third parties may show their concern for fairness and group interests through a preference for punishment. When they are free to choose, third parties show their concern for fairness and group interests as well as the empathy traits through a preference for compensation.

The reputation management is also reflected in the fact that third parties will attempt to avoid the damage to reputation caused by punishment. For example, Rockenbach and Milinski (2011) found that third-party punishers even spend money to hide their punitive behavior. Horita and Takezawa (2014) argued that the presence of audience may be a reference clue for third parties to conduct punishment because they found that the presence of audience only enhances third-party punishment among the people who were not hot tempered but not the people who were hot tempered. It was because third parties who are hot tempered generally do not want their anger to be discovered by others, and due to this, they tend to hide their anger by reducing punishments. Based on the above review, we propose our second hypothesis:

H2: Third parties' preference between punishment and compensation is affected by reputational context. Specifically, under the context of open, which means that the method of third-party fairness maintenance is known by others, if third parties can choose between punishment and compensation, they tend to compensate; if third parties are unable to freely choose, there is no preference between the two methods. Under the context of “secret,” which means that the method of third-party fairness maintenance is not known by others, whether third parties can choose between the two methods or not, there is no preference.

### The Present Study

This study has two focuses: one is the reputation evaluation of fourth parties on third-party fairness maintenance, and the other is the response of third parties to unfair events under the two different reputation contexts of open and secret; these two aspects are both related to whether third parties have the right to free choice. This study includes two component studies. In Study 1, our participants acted as fourth parties and were asked to rate the reputation of third-party fairness maintenance. Two experiments were conducted. In Study 1a, the third parties were not free to choose between punishment and compensation, while in Study 1b, the third parties were free to choose. In Study 2, our participants acted as third parties and were asked to respond to unfair events. Study 2 also included two experiments: in Study 2a, the third parties could not freely choose between the two intervention methods, while in Study 2b, they could freely choose.

## Study 1: Fourth Parties' Reputation Evaluation of Third-Party Fairness Maintenance

### Study 1a: Third Parties Were Not Free to Choose

In this experiment, the participants acted as fourth parties. We presented to them a scenario that involved the dictator game and third-party fairness maintenance. In the scenario, the third parties had to choose between the methods of punishment and compensation as a response to an unfair allocation scheme of the dictator game, and the third parties could not freely choose between the two methods. The participants were asked to evaluate the reputation of the third parties according to the latter's chosen response method. This experiment was concerned about whether there is a difference in the reputation derived from the two methods when a third party is not free to choose between them.

#### Participants

A total of 103 college students were recruited from Shanghai Dianji University (male, 44; age range, 17–23; mean age, *M* ± *SD* = 19.42 ± 1.40). The study was approved by the academic ethics committee of Shanghai Dianji University. All the participants voluntarily participated and signed written informed consent forms. The participants were promised that the experimental results would be kept confidential and would only be used for academic research.

#### Procedure

We presented a scenario that involved the dictator game and third-party fairness maintenance to the fourth-party participants. To increase the authenticity of the experiment, we informed the participants that the scenario had actually happened in our previous experiment. The scenario was as follows: 10 RMB (~US$ 1.55) was allocated between two people, and one of them (dictator) proposed an allocation plan, while the other (recipient) had to accept it. As a result, the dictator allocated 8 RMB (~US$ 1.24) to himself and only 2 RMB (~US$ 0.31) to the recipient.

The third parties needed to respond to this unfair event. The third parties were divided into two treatments; in one, the third parties had to choose between punishment and keep (not intervene), while in the other, the third parties had to choose between compensate and keep.

In both treatments, the third parties were endowed with 10 RMB. If a third party chose to keep, all the 10 RMB would be left to the third party. If the third party chose to punish/compensate, they would have to further decide how much money would be transferred as punishment/compensation. For each RMB to punish/compensate, the dictator/recipient lost/received 1 RMB (~US$ 0.15). The transfer amount of punishment/compensation was sourced from the initial 10 RMB; hence, the choice of punishment/compensation caused some losses for the third party (It should be noted that in the relevant studies, the transfer amount and the lose/gain amount were not equal. Usually, for each RMB to punish/compensate, the dictator/recipient loses/receives 3 RMB. In the present experiment, we were only concerned about what the third parties spend, not what the violators'/victims' lose/gain. In order not to increase the difficulty of arithmetic comprehension, we set the amount spent and the amount lost/gained to be equal). In each treatment, we presented the participants with three choices regarding the third parties, which led to six choice schemes:

1. Choose keep in the punishment treatment (no punishment).
2. Choose punishment in the punishment treatment, with a transfer amount of 3 RMB (~US$ 0.46).
3. Choose punishment in the punishment task, with a transfer amount of 5 RMB (~US$ 0.77).
4. Choose keep in the compensation treatment (no compensation).
5. Choose compensation in the compensation treatment, with a transfer amount of 3 RMB.
6. Choose compensation in the compensation treatment, with a transfer amount of 5 RMB.

We asked the participants to rate the reputation of the third parties of the above six cases from four dimensions: niceness, trustworthiness, group concernedness, and respectability (Barclay, 2006). The Cronbach α was 0.744 in this study. The score range of each dimension was between 1 and 7, and the range of the total score was between 4 and 28. The higher the score, the better the reputation. The experiment was conducted using a computer with the software Eprime 2.0. There were 24 trials; they were presented randomly.

#### Results

**Figure 1:**
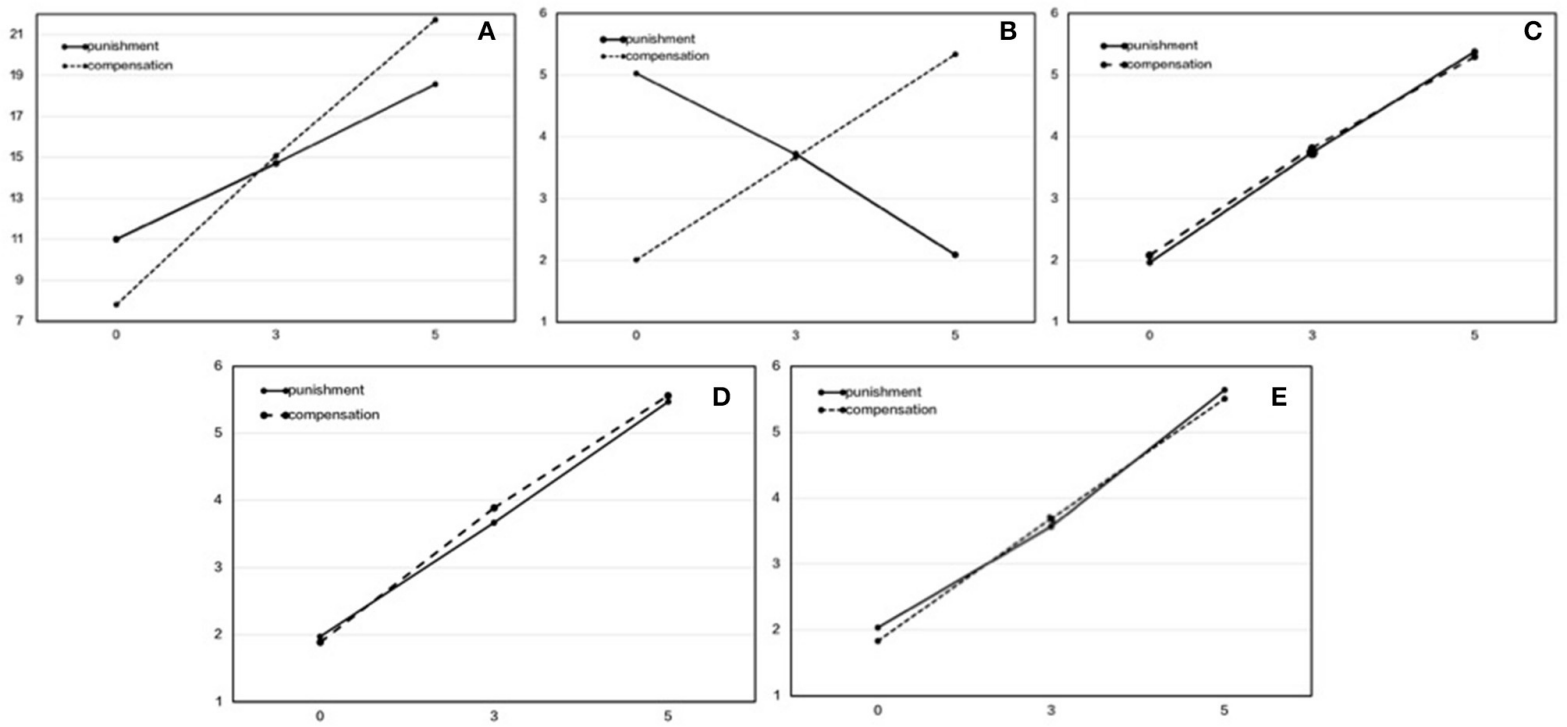
The difference in reputation between punishment and compensation when third parties were not free to choose. The horizontal axis is the transfer amount, and the longitudinal axis is the reputational score. **(A)** Total score. **(B)** Niceness. **(C)** Trustworthiness. **(D)** Group-concernedness. **(E)** Respectability.

If the participants chose “keep,” the transfer amount of punishment/compensation was regarded as 0. We used a 2 (method: punishment, compensation) × 3 (amount: 0, 3, 5) repeated measures analysis of variance (ANOVA) to compare the participants' total scores (see Table 1 and Figure 1). The results showed that the main effect of the method was not significant, *F*_(1, 102)_ = 0.58, *p* = 0.45, *η*^2^_*p*_ = 0.006, which indicates that there was no difference in the reputation between the two methods. The main effect of the amount was significant, *F*_(2, 204)_ = 988.57, *p* < 0.001, η^2^_*p*_ = 0.91. The higher the amount, the higher the total score, and a *post-hoc* test showed that the difference between two amounts was significant. The interaction effect was significant, *F*_(2, 204)_ = 221.56, *p* < 0.001, *η*^2^_*p*_ = 0.69. The simple effect test showed that the total score of punishment was significantly higher than that of compensation when the amount was 0, but compensation was significantly higher than punishment when the amount was 5.

**Table 1:** The reputational score when third parties were not free to choose (*M* ± *SD*).

**Score**	**Punishment**			**Compensation**		
	**0 (keep)**	**3**	**5**	**0 (keep)**	**3**	**5**
Total	11.00 ± 2.10	14.71 ± 2.97	18.57 ± 1.99	7.82 ± 1.80	15.07 ± 2.41	21.72 ± 2.08
Niceness	5.03 ± 1.01	3.72 ± 0.92	2.09 ± 0.97	2.01 ± 0.66	3.67 ± 0.95	5.34 ± 0.88
Trustworthiness	1.97 ± 0.75	3.75 ± 0.86	5.38 ± 0.88	2.08 ± 0.85	3.82 ± 0.93	5.30 ± 0.82
Group-concernedness	1.97 ± 0.86	3.67 ± 0.95	5.47 ± 0.81	1.89 ± 0.69	3.89 ± 1.05	5.56 ± 0.86
Respectability	2.03 ± 0.90	3.57 ± 1.12	5.64 ± 0.77	1.83 ± 0.72	3.69 ± 0.93	5.51 ± 0.86

Previous studies have found that there are differences in specific dimensions of reputation between the two methods (Barclay, 2006). We performed a 2 (method: punishment, compensation) × 3 (amount: 0, 3, 5) repeated measures ANOVA four times to compare the reputational scores for each specific dimension (see Table 1 and Figure 1).

In the niceness dimension, the main effects of the method and amount were both not significant: method, *F*_(1, 102)_ = 0.74, *p* = 0.39, *η*^2^_*p*_ = 0.01; amount, *F*_(2, 204)_ = 3.06, *p* = 0.05, *η*^2^_*p*_ = 0.03. However, the interaction effect between the two was significant, *F*_(2, 204)_ = 571.88, *p* < 0.001, *η*^2^_*p*_ = 0.85. The simple effect test showed that, in punishment, the higher the amount was, the lower the score for niceness was, while in compensation, the higher the amount was, the higher the score for niceness was. This indicates that third-party fairness maintenance was considered to be less nice in the punishment treatment but nicer in the compensation treatment.

For the other three specific dimensions, the main effects of the method were all not significant: trustworthiness, *F*_(1, 102)_ = 0.48, *p* = 0.49, *η*^2^_*p*_ = 0.05; group concernedness, *F*_(1, 102)_ = 1.86, *p* = 0.18, *η*^2^_*p*_ = 0.02; respectability, *F*_(1, 102)_ = 1.60, *p* = 0.21, *η*^2^_*p*_ = 0.02. This indicates that there was no reputational difference in the three specific dimensions between the two methods. The main effects of the amount in the three specific aspects were all significant: trustworthiness, *F*_(2, 204)_ = 572.27, *p* < 0.001, *η*^2^_*p*_ = 0.85; group concernedness, *F*_(2, 204)_ = 741.48, *p* < 0.001, *η*^2^_*p*_ = 0.88; respectability, *F*_(2, 204)_ = 710.02, *p* < 0.001, *η*^2^_*p*_ = 0.85. The higher the amount, the higher the reputational scores in the three dimensions. The interaction effects were all not significant: trustworthiness, *F*_(2, 204)_ = 1.14, *p* = 0.32, *η*^2^_*p*_ = 0.01; group concernedness: *F*_(2, 204)_ = 2.06, *p* = 0.13, *η*^2^_*p*_ = 0.02; respectability, *F*_(2, 204)_ = 3.14, *p* = 3.14, *η*^2^_*p*_ = 0.03. This indicates that whether punishment or compensation, the reputation of third-party fairness maintenance is better than that of keep in these three dimensions. In addition, the higher the transfer amount, the better the reputation one may have.

From the above results, we can see that when third parties cannot choose freely, there will be no difference in the reputation between punishment and compensation, which is consistent with our hypothesis. Whether punishment or compensation, the higher the transfer amount, the better the reputation, but in the niceness dimension, the opposite was true, which indicates that punishment is considered to be a less preferable method to maintain fairness.

### Study 1b: Third Parties Were Free to Choose

Similar to Study 1a, in Study 1b, the participants still acted as the fourth parties. Unlike the first study, the participants were free to choose between punishment and compensation. The experiment was concerned about whether there is a difference in the reputation between the two methods when third parties are free to choose between them.

#### Participants

A total of 107 college students were recruited from Shanghai Dianji University (male, 54; age range, 17–24; mean age, *M* ± *SD* = 19.19 ± 1.41). The study was approved by the academic ethics committee of Shanghai Dianji University. All the participants voluntarily participated and signed written informed consent forms. The participants were promised that the experimental results would be kept confidential and that they would only be used for academic research.

#### Procedure

The participants were presented with a scenario which was similar to Study 1a, and they had to choose a respond method. Unlike Study 1a, there was only one case for the third parties; they could choose between keep (not intervene), punishment, and compensation, which means that the third parties were free to choose between compensation and punishment. We presented the participants with five choices regarding third parties:

1. Choose keep (non-punishment and non-compensation).
2. Choose punishment, with a transfer amount of 3 RMB.
3. Choose punishment, with a transfer amount of 5 RMB.
4. Choose compensation, with a transfer amount of 3 RMB.
5. Choose compensation, with a transfer amount of 5 RMB.

Similar to Study 1a, the participants had to rate the reputation in the five subdimensions (Barclay, 2006). The Cronbach α of the five subdimensions in the study was 0.767. There were 20 trials in this study, which were presented randomly.

#### Results

We treated the choice of “keep” as non-intervention and treated the punishment of 3 RMB and 5 RMB as punishment and the compensation of the two amounts as compensation. Thus, the third parties were divided into three treatments (keep, punishment, and compensation). We used a repeated measures ANOVA to compare the total reputational scores of the three treatments. The total reputational score of punishment/compensation was the average score of the two amounts. The results showed that the difference was significant, and a *post-hoc* test showed that the compensation was higher than punishment and keep, and punishment was significantly higher than compensation. Following this, we conducted a repeated measures ANOVA to compare the reputational score of the four specific dimensions in the three treatments. The results showed that the differences in the four specific dimensions were all significant. In the niceness dimension, compensation was higher than punishment and keep, and keep was higher than punishment, while in the other three dimensions, compensation was higher than punishment and keep, and punishment was significantly higher than keep (see Table 2).

**Table 2:** The difference in the reputational scores when third parties were free to choose (*M* ± *SD*).

**Score**	**Keep**	**Punishment**	**Compensation**	***F***_**(2, 212)**_	$\eta_{\text{p}}^{2}$
Total	11.58^c^ ± 3.40	19.02^b^ ± 2.02	24.34^a^ ± 1.82	1021.84^***^	0.91
Niceness	5.17^b^ ± 0.99	3.70^c^ ± 0.81	6.01^a^ ± 0.54	270.54^***^	0.72
Trustworthiness	2.12^c^ ± 1.09	5.18^b^ ± 0.72	6.05^a^ ± 0.64	828.04^***^	0.89
Group-concernedness	2.25^c^ ± 1.21	4.83^b^ ± 0.64	6.13^a^± 0.56	649.35^***^	0.86
Respectability	2.04^c^ ± 1.19	5.32^b^ ± 0.72	6.14^a^ ± 0.60	773.22^***^	0.88

*** *p < 0.001. a was higher significantly than b and c, and b was higher significantly than c*.

**Figure 2:**
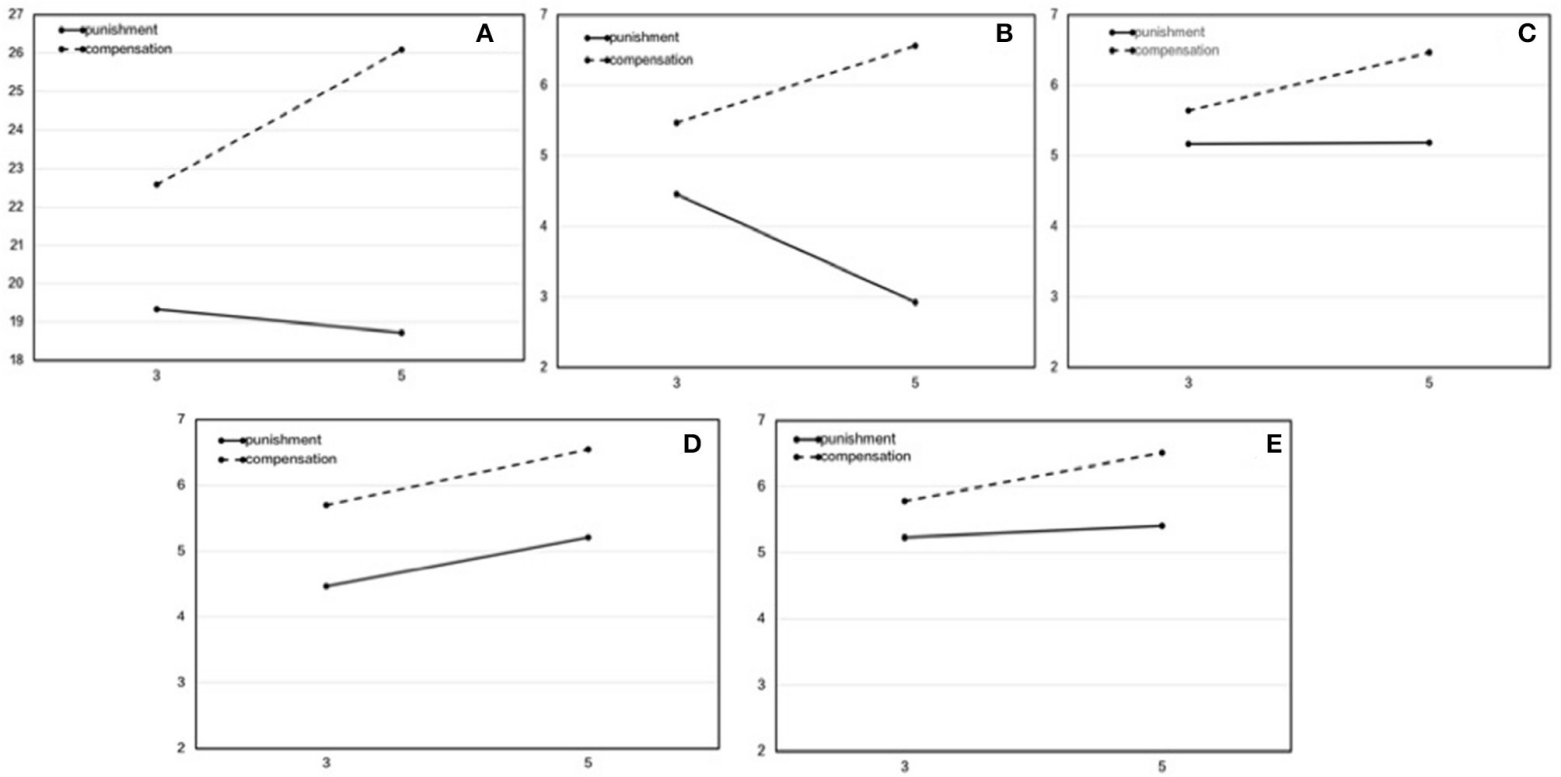
The difference in reputation between punishment and compensation when third parties were free to choose. The horizontal axis is the transfer amount, and the longitudinal axis is the reputational score. **(A)** Total score. **(B)** Niceness. **(C)** Trustworthiness. **(D)** Group-concernedness. **(E)** Respectability.

To further investigate the reputational difference of punishment and compensation between the different amounts, we used a 2 (amount: 3, 5) × 2 (method: punishment, compensation) repeated measures ANOVA five times to compare the differences in the total scores and the four specific dimensions (see Table 3 and Figure 2).

**Table 3:** The reputational scores of different transfer amounts for punishment and compensation (*M* ± *SD*).

**Score**	**Punishment**		**Compensation**	
	**3**	**5**	**3**	**5**
Total	19.33 ± 2.49	18.73 ± 2.93	22.58 ± 3.05	26.09 ± 1.48
Niceness	4.46 ± 1.07	2.93 ± 1.11	5.47 ± 0.93	6.56 ± 0.54
Trustworthiness	5.17 ± 0.96	5.19 ± 1.03	5.64 ± 0.94	6.47 ± 0.68
Group-concernedness	4.47 ± 0.97	5.21 ± 1.00	5.70 ± 0.86	6.55 ± 0.59
Respectability	5.23 ± 1.09	5.41 ± 0.95	5.78 ± 1.14	6.51 ± 0.57

Regarding the total scores, the main effect of the method was significant, *F*_(1, 106)_ = 503.07, *p* < 0.001, *η*^2^_*p*_ = 0.83, and compensation was higher than punishment. This indicates that the reputation of compensation is better than that of punishment. The main effect of the amount was significant, *F*_(1, 106)_ = 53.69, *p* < 0.001, *η*^2^_*p*_ = 0.34, and the reputational score for 5 RMB was higher than that for 3 RMB. This indicates that the higher the transfer amount, the better the reputation. The interaction effect was significant, *F*_(1, 106)_ = 62.75, *p* < 0.001, *η*^2^_*p*_ = 0.37. A simple effect test showed that, in punishment, the reputational score for 3 RMB was higher than that for 5 RMB, while in compensation, the reputational score for 5 RMB was higher than that for 3 RMB.

In the niceness dimension, the main effect of the method was significant, *F*_(1, 106)_ = 590.34, *p* < 0.001, *η*^2^_*p*_ = 0.85. Compensation was higher than punishment. The main effect of the amount was significant, *F*_(1, 106)_ = 7.43, *p* = 0.007, *η*^2^_*p*_ = 0.07. In addition, 3 RMB was higher than 5 RMB. The interaction effect was significant, *F*_(1, 106)_ = 207.46, *p* < 0.001, *η*^2^_*p*_ = 0.66. A simple effect analysis showed that, in punishment, the reputational score for 3 RMB was higher than that for 5 RMB, while in compensation, that for 5 RMB was higher than that for 3 RMB.

In the other three dimensions, the main effects of the method were all significant, and the scores for compensation were higher than those for punishment: trustworthiness, *F*_(1, 106)_ = 104.29, *p* < 0.001, *η*^2^_*p*_ = 0.50; group concernedness, *F*_(1, 106)_ = 232.86, *p* < 0.001, *η*^2^_*p*_ = 0.69; respectability, *F*_(1, 106)_ = 96.95, *p* < 0.001, *η*^2^_*p*_ = 0.48. The main effects of the amount were all significant, and the scores for 5 RMB were higher than those for 3 RMB: trustworthiness, *F*_(1, 106)_ = 26.84, *p* < 0.001, *η*^2^_*p*_ = 0.20; group concernedness, *F*_(1, 106)_ = 91.21, *p* < 0.001, *η*^2^_*p*_ = 0.46; respectability, *F*_(1, 106)_ = 28.30, *p* < 0.001, *η*^2^_*p*_ = 0.21. The interaction effect of trustworthiness was significant, *F*_(1, 106)_ = 24.01, *p* < 0.001, *η*^2^_*p*_ = 0.19. The simple effect test showed that, in punishment, the difference was not significant between 3 RMB and 5 RMB, while in compensation, that for 5 RMB was significantly higher than that for 3 RMB. The interaction of group concernedness was not significant, *F*_(1, 106)_ = 0.41, *p* = 0.53, *η*^2^_*p*_ = 0.004. The interaction effect of respectability was significant, *F*_(1, 106)_ = 7.30, *p* = 0.008, *η*^2^_*p*_ = 0.064. The simple effect test showed that, in punishment, the difference was not significant between 3 RMB and 5 RMB, while in compensation, that for 5 RMB was significantly higher than that for 3 RMB.

From the above results, we can see that no matter the total score or subdimensions, the reputation of compensation is better than that of punishment, which indicates that compensation can lead to a better reputation when third parties are free to choose between the two methods. This is different from Study 1a but consistent with our hypothesis. Moreover, compared with high punishment, low punishment is considered to be nicer, which indicates that punishment is considered to be less nice. This is consistent with the findings of Study 1a.

## Study 2: Third Parties' Response to Unfair Events

### Study 2a: Third Parties Were Not Free to Choose

In this study, the participants acted as the third parties. We presented them with unfair allocation schemes of the dictator game, and there were two reputational contexts: open and secret. In the face of unfair events, the third parties either chose to respond between keep and punish or between keep and compensate. We were concerned about whether reputational contexts would affect the choice between the two methods when third parties are not free to choose.

#### Participants

A total of 225 college students were recruited from Shanghai Dianji University (male, 126; age range, 16–24; mean age, *M* ± *SD* = 19.86 ± 1.75). The study was approved by the academic ethics committee of Shanghai Dianji University. All the participants voluntarily participated and signed written informed consent forms. The participants were promised that the experimental results would be kept confidential and that they would only be used for academic research.

#### Procedure

The experiment was carried out using a computer with the software Eprime 2.0. The participants were taken to a separate room where they were told that there were two other people playing the dictator game in two other rooms: one person was the dictator, and the other was the recipient. In the game, the dictator had to allocate 10 RMB between himself and the recipient. There were nine allocation schemes for the dictator to choose: 9:1 (i.e., the dictator was allocated 9 RMB and the recipient 1 RMB), 8:2, 7:3, 6:4, 5:5, 4:6, 3:7, 2:8, and 1:9. The dictator's choice was shown to the participants via a computer, and we presented five allocation schemes to the participants: 9:1, 8:2, 7:3, 6:4, and 5:5. However, the dictator game did not, in fact, occur. The five presented schemes had been preset via the computer programs, but the participants thought that they had been made by a dictator. The participants were randomly divided into four experimental treatments of 2 (reputational context: open, secret) × 2 (method: punishment, compensation): open punishment, open compensation, secret punishment, and secret compensation.

Under the open context, the participants were informed that their choices would be open to other participants when the experiment ended; however, this did not occur, but the participants believed it would. Under the secret context, the participants were informed that their choices would be kept secret. In the punishment condition, the participants chose between keep and punishment, while in the compensation condition, they chose between keep and compensation.

The participants were endowed with a basic fee of RMB 10 (~US$1.55) and another additional 50 money units (MUs); each MU corresponded to RMB 0.1 (~US$0.016). If the participants chose keep, all the basic fees and additional MUs, a total of RMB 15 (~US$2.33), would be left to themselves. If they chose to punish/compensate, they would further select how much MUs would be transferred to punish/compensate. There were four transfer schemes that participants could select: 5, 10, 15, and 20. For each MU that would be transferred as a punishment/compensation, the dictator/recipient would lose/gain 3 MUs. The transferred MUs were obtained from the original 50 MUs. There were five trials that were presented randomly. We choose one trial randomly and paid the test fees according to the participants' selections. The test fee was calculated as follows: 10 + (50-transfer MUs)/10.

#### Results

Previous studies have found that in the face of fair allocation, third parties generally chose keep (Hu et al., 2015; Liu Y. et al., 2019). In this experiment, for the 5:5 allocation scheme, all the participants chose keep. Based on previous research, we only analyzed four unfair schemes (Hu et al., 2015; Liu Y. et al., 2019).

The choice of keep means non-intervention, and the choice of punish and compensate should be treated as an intervention. Thus, the expected rate for keep and intervention were all 0.5. To investigate whether third parties would be inclined to intervene in unfair events under four experimental conditions, we used a single sample *t*-test to compare the intervention rates with the rate of 0.5 (see Table 4). Intervention rates were calculated as the intervention times divided by 4 (Liu Y. et al., 2019). The results showed that in the four conditions, the intervention rates were all significantly higher than 0.5: secret punishment, *t*_(224)_ = 3.34, *p* = 0.002; secret compensation, *t*_(224)_ = 2.56, *p* = 0.01; open punishment, *t*_(224)_ = 6.65, *p* < 0.001; and open compensation, *t*_(224)_ = 8.14, *p* < 0.001. This indicates that, when faced with unfair events, third parties are inclined to intervene.

**Table 4:** Transfer rates and amounts when third parties were not free to choose (*M* ± *SD*).

	**Secret-** **punishment**	**Secret-** **compensation**	**Open-** **punishment**	**Open-** **compensation**
Rate	0.67 ± 0.36	0.61 ± 0.30	0.75 ± 0.29	0.75 ± 0.25
Amount	8.53 ± 5.16	7.72 ± 4.23	10.35 ± 4.73	10.04 ± 3.61

**Figure 3:**
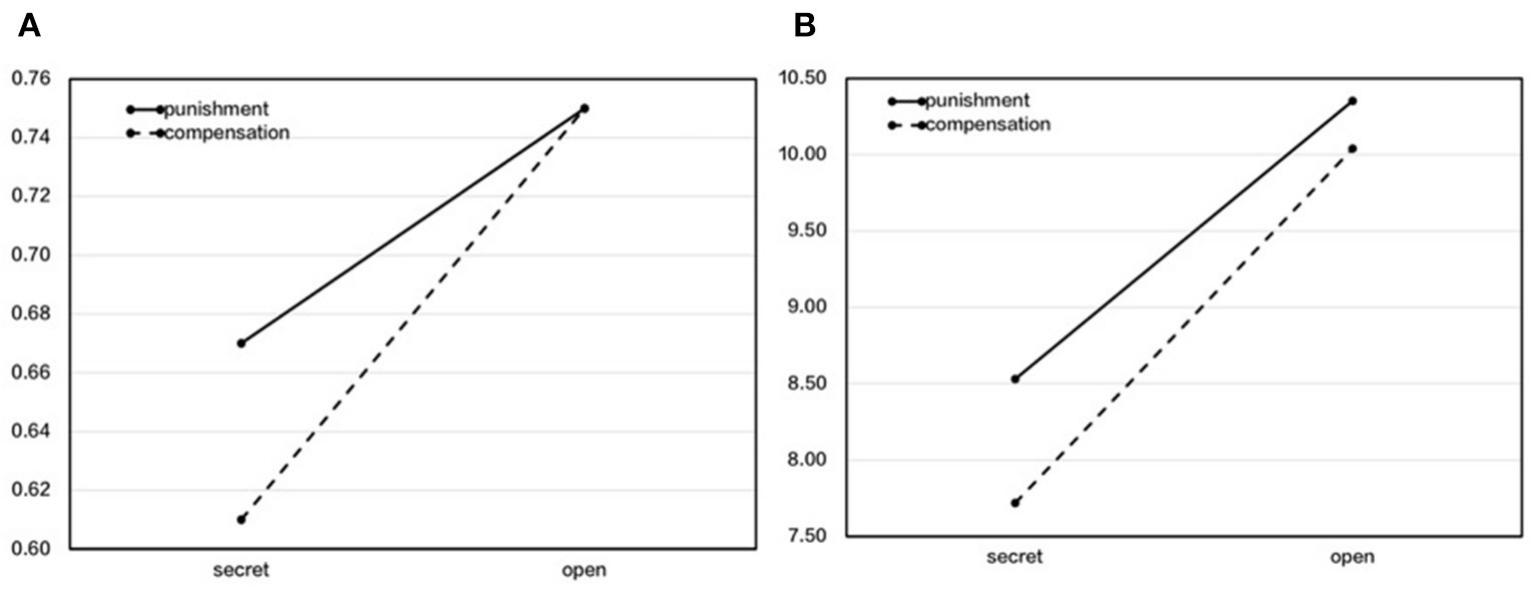
The difference in transfer rates and amounts between punishment and compensation under the contexts of secret and open when participants were not free to choose. **(A)** Transfer rate. **(B)** Transfer amount.

To investigate the effect of the reputational contexts and the intervention method on transfer rates, we used a two-way ANOVA of 2 (reputational context: secret, open) × 2 (method: punishment, compensation) to compare the transfer rates in the four experimental treatments (see Figure 3). The results showed that the main effect of the reputational context was significant, *F*_(1, 221)_ = 8.04, *p* = 0.005, *η*^2^_*p*_ = 0.04, and that of open was significantly higher than that of secret. The main effect of the method was not significant, *F*_(1, 221)_ = 0.50, *p* = 0.48, *η*^2^_*p*_ = 0.002. Furthermore, the interaction effect was not significant, *F*_(1, 221)_ = 0.64, *p* = 0.42, *η*^2^_*p*_ = 0.003.

We then used a same two-way ANOVA to compare the transfer amounts in the four experimental treatments (see Figure 3). The results showed that the main effect of the reputational context was significant, *F*_(1, 224)_ = 12.11, *p* = 0.001, *η*^2^_*p*_ = 0.05, and that of open was significantly higher than that of secret. The main effect of the method was not significant, *F*_(1, 224)_ = 0.90, *p* = 0.35, *η*^2^_*p*_ = 0.004. The interaction effect was not significant, *F*_(1, 224)_ = 0.18, *p* = 0.68, *η*^2^_*p*_ = 0.001.

Based on the above results, we can see that, under the open context, which is good for one's reputation, third-party fairness maintenance will increase, while under the secret context, it may decrease. In addition, we can also conclude that when the third party cannot choose freely, there is no preference between the two methods regarding third-party fairness maintenance.

### Study 2b: Third Parties Were Free to Choose

In this experiment, the participants still acted as the third parties. The participants were presented with unfair results of the dictator game. Unlike Study 2a, in this experiment, when faced with unfair events, third parties could choose among keep, punish, and compensate. There were still two reputational treatments: secret and open. We were concerned about whether reputational contexts would affect third parties' choice between the two methods when they are free to choose.

#### Participants

A total of 126 college students were recruited from Shanghai Dianji University (male, 67; age range, 17–24; mean age, *M* ± *SD* = 20.11 ± 1.61). The study was approved by the academic ethics committee of Shanghai Dianji University. All the participants voluntarily participated and signed written informed consent forms. The participants were promised that the experimental results would be kept confidential and that they would only be used for academic research.

#### Procedure

Similar to Study 2a, the participants were asked to choose a method to respond to the unfair schemes of the dictator game, and the participants were divided into two experimental treatments: secret and open. Unlike Study 2a, faced with the game result, participants could either choose keep, punishment, or compensation as a response. All other settings were similar to that applied in Study 2a.

#### Results

Similar to Study 2a, faced with the result of 5:5, all the participants chose keep; thus, we solely analyzed the four unfair schemes (Hu et al., 2015; Liu Y. et al., 2019). We treated punishment and compensation as intervention, and the expected rate of the intervention was 0.5. To investigate whether third parties are inclined to intervene in unfair events, we used a single sample *t-*test to compare the intervention rates with the rate of 0.5. The results showed that in the two experimental treatments, the intervention rates were all significantly higher than 0.5 (secret: *M* ± *SD* = 0.54 ± 0.25, *t* = 1.26, *p* = 0.04; open: *M* ± *SD* = 0.71 ± 0.21, *t* = 8.01, *p* < 0.001). This indicates whether open to others or not, third parties are inclined to intervene in unfair events.

**Figure 4:**
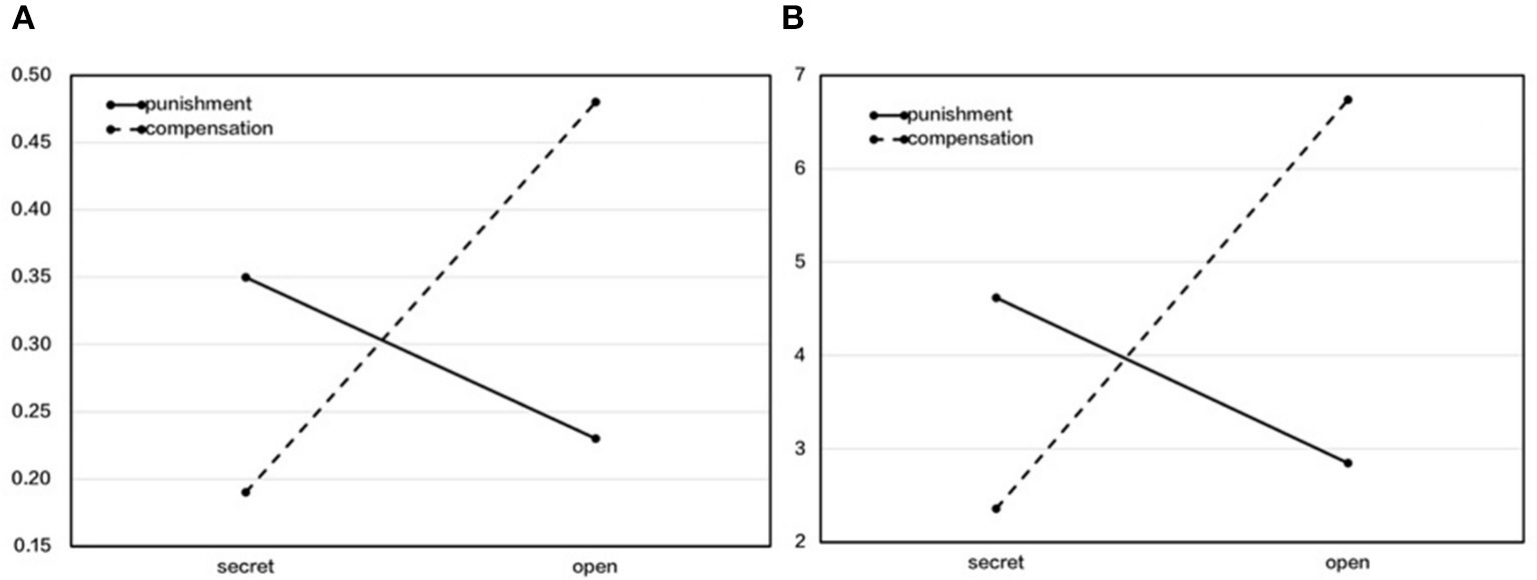
The difference in transfer rates and amounts between punishment and compensation under the contexts of secret and open when participants were free to choose. **(A)** Transfer rate. **(B)** Transfer amount.

To analyze the effect of the reputational context and the method on third-party fairness maintenance, we used a mixed two-way ANOVA of 2 (between, reputational contexts: secret, open) × 2 (within, method: punishment, compensation) to compare the intervention rates (see Table 5 and Figure 4). The results showed that the main effect of the method was not significant, *F*_(1, 123)_ = 0.69, *p* = 0.41, *η*^2^_*p*_ = 0.006. The main effect of the reputational context was significant, *F*_(1, 123)_ = 17.60, *p* < 0.001, *η*^2^_*p*_ = 0.12, and that of open was significantly higher than that of secret. The interaction effect was significant, *F*_(1, 123)_ = 20.18, *p* < 0.001, *η*^2^_*p*_ = 0.14. The simple effect test showed that under the secret context, the punishment rate was higher than that of compensation, while under the open context, the compensation rate was higher than that of punishment.

**Table 5:** Transfer rates and amounts when third parties were free to choose (*M* ± *SD*).

	**Secret-** **punishment**	**Secret-** **compensation**	**Open-** **punishment**	**Open-** **compensation**
Transfer rate	0.35 ± 0.25	0.19 ± 0.23	0.23 ± 0.30	0.48 ± 0.35
Transfer amount	4.62 ± 3.88	2.36 ± 3.44	2.85 ± 3.87	6.74 ± 5.91

Following this, we used the same mixed two-way ANOVA to compare the transfer amounts (see Table 5 and Figure 4). The results showed that the main effect of the method was not significant, *F*_(1, 123)_ = 1.34, *p* = 0.24, *η*^2^_*p*_ = 0.01. The main effect of the reputational context was significant, *F*_(1, 123)_ = 12.51, *p* = 0.001, *η*^2^_*p*_ = 0.092, and the transfer amount of open was significantly higher than that of secret. The interaction effect was significant, *F*_(1, 123)_ = 19.86, *p* < 0.001, *η*^2^_*p*_ = 0.14. A simple effect test showed that under the secret context, the punishment amount was significantly higher than that of compensation, while under the open context, the compensation amount was significantly higher than that of punishment.

From the above results, we can see that third-party fairness maintenance increased under the open context, and this was consistent with the findings of Study 2a. What was inconsistent, which was also more important, was that we found an interaction effect. Under the secret context, the third parties tended to punish, while under the open context, they tended to compensate. This indicates that third parties' preference between punishment and compensation was mediated by the reputational context, and they conduct reputational management according to reputational contexts.

## Discussion

Third-party fairness maintenance will lead to a dilemma of interests between a group and an individual. Indirect reciprocity theory resolves this dilemma and provides a sound explanation regarding the motivation for the third-party fairness maintenance, and reputational benefits play an important role in this regard. The present study verified the effect of reputational benefits on third-party fairness maintenance through the dual perspectives of fourth parties and third parties.

### The Interaction Focusing on the Reputation Between Fourth Parties and Third Parties

This study present us with an interesting interaction between third and fourth parties. Fourth parties evaluate the reputation through the choices of the third parties and the option, while the third party adjusts its choice according to the option and the reputational context to win a good reputation. There is a Chinese proverb: “the road is high one-foot, the evil spirit is high one unit of length,” which is a vivid description of this interaction.

Reputation is a precious social resource, which can only be given to third parties who are truly altruistic. Through this, the interests of a group and third-party individuals could be guaranteed. The third parties' choice between punishment and compensation and whether they were free to choose provides a basis for the reputational evaluations of fourth parties. Third-party fairness maintenance includes two aspects: the willingness to intervene and the way to intervene. The former indicates whether a third party tends to intervene in unfair events, while the latter indicates the preference between the two methods. When third parties are free to choose, whether punishment or compensation, this means that the third parties have the willingness to maintain fairness, and there would be no reputational difference between the two methods. When third parties are free to choose, their choice can reflect the third parties' preference between the two ways and the true motivation behind it, and the reputation of compensation would be better than that of punishment. For third parties, when they are not free to choose, they need to show their concern for fairness and group interests through intervening in unfair events, while when they are free to choose, they need to show a preference for compensation and signal to others that they are concerned about both fairness and compassion.

Fourth parties typically try to understand the true motivation behind third-party fairness maintenance, while third parties try to cover up the punishment that is damaging their reputation and show the compensation that is conducive to their reputation. Fourth parties and third parties seem to be engaged in a contest. A similar phenomenon has been found in a study by Rockenbach and Milinski (2011). In their study, participants participated in the PGG with punishment, and observers were set up. They found that the observers would rather spend money to understand the players' contributions in the PGG and their punishment decisions. Players are also willing to spend to hide their punishment decisions but are also willing to open their contributions in the PGG.

In real social life, moral behaviors of different nature are often treated differently. We will protect the privacy of moral behaviors that is similar to punishment such as reporting but propagandize moral behaviors, which are similar to compensation such as donation. The whistleblowers may be retaliated, and reputational benefits may not be able to make up for the loss caused by retaliation. The best setting for a whistleblower is to get monetary reward and give up the reputational benefits. This is consistent with the strategy of third-party fairness maintenance: choose punishment under the secret context, but choose compensation under the open context.

### Third-Party Fairness Maintenance Has a Dual Feature of Altruism and Selfishness

As a kind of prosocial behavior, third-party fairness maintenance has been labeled as moral. However, recent studies had shown that human's prosocial behaviors are not only motivated by social norms but also by personal norms (Capraro and Perc, 2021). Social norm is an external standard of moral behavior, while the personal norm is an internal standard, which will be presented in the form of moral framework (Jordan and Rand, 2020; Capraro et al., 2021). The affections of observers on the two kinds of norms are different. It has been found that people's public behavior is driven by social norms (Schram and Charness, 2015), but the private behavior is driven by personal norms (Capraro and Rand, 2018).

The present study found that in the face of unfair events, the third party will tend to intervene, but it is still affected by observers. Under the secret context, the intervention rate of third parties was higher than the theoretical rate of 0.5 and tended to punish. However, under the open context, the intervene rate of third parties was higher and tended to compensate. This indicate that third-party fairness maintenance may be driven by both social norms and personal norms. In the previous studies, both the social norms and personal norms are all altruistic. However, in this study, we found that the individual norms of third-party fairness maintenance have a consideration of self-interest.

Although it has a feature of selfish, we should not belittle the moral value of third-party fairness maintenance. Indirect reciprocity is not only the result of third-party fairness maintenance but also the motivation behind it. Third-party fairness maintenance brings indirect reciprocity, which in turn stimulates third-party fairness maintenance. In this way, a virtuous circle is formed, so that third-party fairness maintenance can be sustained, and the group interest, as well as fairness morality, can also be guaranteed.

### Third-Party Punishment Is a Double-Edged Sword

In this study, we found that the punisher is considered to be less nice. Punishment has two sides: it can be both loved and feared (Gordon et al., 2014; Kroupa, 2014). Previous studies have found that punishers can win some good reputation, which, however, may not necessarily translate into practical benefits (Ozono and Watabe, 2012). In addition, the effect of punishment on promoting cooperation has also been questioned. Mulder et al. (2006) had found that when the third-party punishment was added, the cooperation of players in PGG would increase, but when the punishment was withdrawn, the cooperation would become less. Other studies have made similar observations (Wang and Chen, 2011; Cui et al., 2017). Sometimes, the violator even bribe the third-party punishers to avoid being punished (Liu L. et al., 2019). Once corruption occurs, the third-party punishers will not only fail to promote cooperation but will undermine it.

Since the effect of third-party punishment in realizing indirect reciprocity and promoting cooperation is uncertain, what is its significance? For third-party punishment, we should distinguish between the existence and use of it. The existence of a third-party punishment mechanism can provide deterrence to group members and reduce their violations to promote cooperation, but the implementation of it may reduce cooperation. Therefore, a third-party punishment mechanism could exist, but the third party should use it less (Chen et al., 2014; Kroupa, 2014). Even if punishment is imposed, the mild punishment is more appropriate than the intensive punishment (Chen et al., 2015). This is akin to police armed with guns, who can deter criminals, but rarely fire; even when firing, such guns are usually limited in power and are just used to stop crimes but not to kill criminals. In addition, a more reasonable third-party punishment mechanism should be adopted. For example, the use of collective punishment rather than individual punishment can avoid being retaliated and reduce the cost of punishment (Sigmund et al., 2011). Besides, sharing punishment responsibility based on probability can also reduce costs (Chen et al., 2014). The reduction in cost is beneficial to the sustain of punishment (Wang et al., 2020).

Although one's reputation can be negative, people's real preference may be punishment. In Study 2b, we found that the third parties tended to choose punishment under the secret context but tended to choose compensation under the open context, indicating that the real reaction of third parties to unfair events is punishment, while compensation is just a strategic response to win a good reputation. Liu Y. et al. (2019) had found that under time pressure, the priority response of third parties is punishment; thus, they argued that people's instinctive response to unfair events is anger and punishment. In addition, in another study by Liu et al. (2017), it was found that third parties tend to punish in a “gain” context but compensate in a “loss” context. The present study was conducted under the gain context; hence, under the secret context, the real reaction of the third parties may be punishment.

### Limitation

In the present study, the third parties either chose between keep and punish/compensate or between keep, punish, and compensate, which was a single choice; that is, they could only respond to unfair events in one way. In fact, the third parties could have another choice, in what is known as “double choice,” which means that the third parties could choose punishment and compensation at the same time. A double choice includes both anger at the violator and empathy for the victim; hence, the motivation behind such a choice would be more complicated. Thus, this raises a series of questions: How do fourth parties understand the motivation of double election, and what reputation evaluation would be given to this motivation? Do third parties have a preference between single choice and double choice? To answer these questions, double choices should be considered in future research.

## Conclusion

The difference in reputations of the third-party punishment and compensation is modulated by whether one is free to choose between the two methods. Specifically, when a third party is not free to choose, there is no reputational difference between the two methods, and the reputation of the two methods are all better than that of non-intervention. However, when there is the freedom to choose, the reputation of compensation is better than that of punishment, and the reputation of the two methods is also better than that of non-intervention.

To gain a good reputation, a third party will conduct reputational management through the choice between punishment and compensation, and the choice should also be mediated by whether there is the freedom to choose as well as the reputational context. Specifically, when there is no freedom to choose between the two methods, punishment and compensation under the open context occur more compared with the secret context, and there is no preference for the third party between the two methods. When there is the freedom to choose between the two methods, third-party punishment and compensation under the open context also occur more compared with the secret context; third parties prefer punishment under the secret context, while prefer compensation under the open context.

## Data Availability Statement

The datasets presented in this study can be found in online repositories. The names of the repository/repositories and accession number(s) can be found at: The datasets for this study can be found in the “figshare.” URL: https://doi.org/10.6084/m9.figshare.14139971.v1.

## Ethics Statement

The studies involving human participants were reviewed and approved by the Academic Ethics Committee of Shanghai Dianji University. Written informed consent to participate in this study was provided by the participants' legal guardian/next of kin.

## Author Contributions

ZL conceived the paper, ran statistical analyses, and contributed to the manuscript. GH conceived the paper and contributed to the manuscript. LX conducted the experiments. QL contributed to the manuscript. All authors contributed to the article and approved the submitted version.

## Conflict of Interest

The authors declare that the research was conducted in the absence of any commercial or financial relationships that could be construed as a potential conflict of interest.
